# Professional Exercise Recommendations for Healthy Women Who Are Pregnant: A Systematic Review

**DOI:** 10.1089/whr.2021.0077

**Published:** 2021-09-20

**Authors:** Lauren R. Rudin, Leslie Dunn, Kaitlyn Lyons, Jill Livingston, Molly E. Waring, Linda S. Pescatello

**Affiliations:** ^1^ Department of Kinesiology, University of Connecticut, Storrs, Connecticut, USA.; ^2^ Central Connecticut State University, New Britain, Connecticut, USA.; ^3^ Wesleyan Library, Wesleyan University, Middletown, Connecticut, USA.; ^4^ Department of Allied Health Sciences, University of Connecticut, Storrs, Connecticut, USA.; ^5^ Institute for Collaboration on Health, Intervention, and Policy, University of Connecticut, Storrs, Connecticut, USA.

**Keywords:** guidelines, physical activity, pregnancy, prenatal, professional society

## Abstract

***Background:*** Exercise in pregnancy favorably affects maternal and fetal outcomes, yet only 50% of women receive exercise guidance during prenatal care and 15% are told to stop exercising. Reasons for clinician reluctance to recommend exercise include safety concerns and ambiguity of recommendations. To better inform clinicians, this systematic review assembled a consensus exercise prescription (ExRx) for healthy pregnant women framed by the Frequency, Intensity, Time, and Type (FITT) principle.

***Methods:*** In April 2021, PubMed, Scopus, SPORTDiscus, Cumulative Index of Nursing and Allied Health Literature (CINAHL), and Cochrane databases were searched. Reports were eligible if: (1) targeted healthy pregnant women, (2) framed the ExRx by the FITT, and (3) published by a professional society from 2000 to 2021 in English. The Appraisal of Guidelines for Research and Evaluation II tool assessed risk of bias.

***Results:*** Twelve reports of poor to good quality were included. Nine societies conducted systematic reviews, but only three provided a detailed, transparent description of the review conducted. Although the FITT varied, the most common was most days of the week, moderate intensity, 30 minutes/session to accumulate 150 minutes/week, and aerobic, resistance, and flexibility exercise with three societies advising neuromotor exercise. All professional societies specified activities to avoid and eight societies included contraindications to exercise.

***Conclusions:*** This systematic review produced a consensus ExRx for healthy pregnant women to better inform clinicians about advising their patients to exercise during pregnancy. Future research is needed to determine the upper limits of exercise while pregnant and provide better informed guidance relating to safety concerns for women who are pregnant.

## Introduction

Regular physical activity has a positive effect on the mind and body of pregnant women and on maternal and fetal outcomes, including a lower incidence of excessive gestational weight gain, postpartum weight retention, gestational diabetes mellitus, preeclampsia, preterm or Cesarean birth, and having an infant with low birth weight.^1–7^ Despite these numerous benefits, many women who are pregnant become less physically active or stop exercising altogether.^8^ Possible reasons for the decreased levels of physical activity during pregnancy are that only half of women receive exercise guidance during prenatal care meetings and 15% report being told to stop exercising by their prenatal care team.^8^ Research suggests that some prenatal care providers do not feel comfortable providing advice on physical activity during pregnancy; one study found that 68% of health care providers report feeling “comfortable” or “very comfortable” providing such advice to their pregnant patients.^9^ Other reasons for the reported decreased rates of physical activity during pregnancy compared with prepregnancy include a lack of knowledge about exercise, subsequent safety concerns and fears of exercising during pregnancy, and the long-held mistaken notion that exercise can be harmful to both the mother and infant.^1,10–15^ In response to these misguided beliefs, in 2002 the American College of Obstetrics and Gynecology (ACOG) issued a position statement that pregnancy is a unique time for behavior modification and should no longer be a time for bed rest, and ACOG has expanded on these new findings in subsequent committee opinions.^16^

Professional organizations^3–7,17–24^ world-wide have released guidelines for exercise during pregnancy, but they do not unanimously agree on the frequency, intensity, duration, or type of exercise program that is best or the foundation for developing an exercise prescription (ExRx). The methodologies utilized to generate recommendations vary as well, all of which may contribute to misinformation and lack of recommending exercise during prenatal care visits.

An ExRx is the process whereby an individualized physical activity program is designed using the Frequency (how often), Intensity (how hard), Time (how long), and Type (what kind) or FITT principle of ExRx.^25^ Both constitutional and methodological discrepancies among the various exercise recommendations for women who are pregnant highlight the need for consensus physical activity guidelines to properly inform prenatal care providers and pregnant women about an appropriate ExRx for healthy pregnancies. This systematic review aims to examine the existing professional society exercise recommendations for healthy women who are pregnant and arrive at a consensus FITT ExRx. In addition, we will compile important special considerations that may impact the ability to exercise during pregnancy, such as unique exercise testing, preparticipation screening procedures and pregnancy-related contraindications.

## Materials and Methods

### Information sources and search strategy

**FIG. 1.**
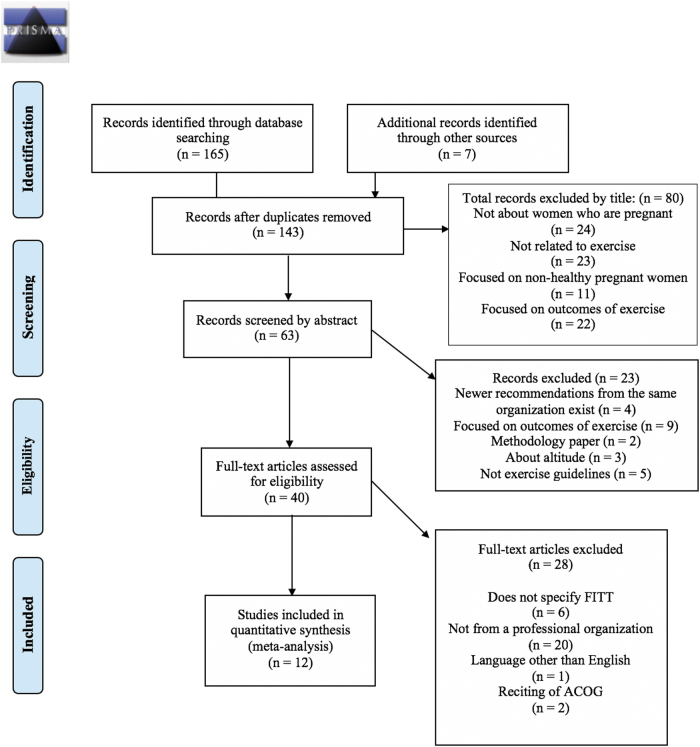
PRISMA diagram for study selection. PRISMA, Preferred Reporting Items for Systematic Reviews and Meta-Analyses.

This systematic review was conducted according to the Preferred Reporting Items for Systematic Reviews and Meta-Analyses (PRISMA) guidelines.^26,27^ We searched PubMed, Scopus, SPORTDiscus, Cumulative Index of Nursing and Allied Health Literature (CINAHL), and the Cochrane library from inception to April 1, 2021. We also hand-searched reference lists of articles chosen for full-text review to identify additional qualifying studies. Keywords searched included words related to pregnancy, exercise, recommendations, and professional statements. See Figure 1 for the selection process and Supplementary Data for the search strategy. Institutional Review Board approval was not required for this study.

### Eligibility criteria

Publications were eligible for inclusion if they: (1) were intended for healthy pregnant women, (2) were published by a professional society defined as a national-level health-related professional organization interested in pregnancy/midwifery, exercise, physical activity, and/or sports medicine that published a committee opinion, position statement, consensus statement, guidelines, and/or handouts/packets for clinical utilization and/or distribution, (3) included the FITT of the ExRx, and (4) were published in English from January 1, 2000 to April 1, 2021. We only included articles published in this timeframe to incorporate recommendations based on the most recent evidence. Articles were excluded if they: (1) have been replaced by a more recent version of the guidelines from the same professional society, (2) were designed for use by women with gestational diabetes mellitus, preeclampsia, or long-term health conditions or nonpregnant women, or (3) included diet.

### Data extraction

The database search was performed in consultation with a medical librarian (J.L.). Two investigators (L.D. and L.R.R.) screened all titles (*n* = 143), abstracts (*n* = 63), and full-text articles (*n* = 40) of all potentially qualifying reports that yielded 12 qualifying reports (Fig. 1). Any disagreements between investigators were resolved *via* discussion and consensus. Two authors (L.D. and L.R.R.) extracted relevant information from each report to complete the evidence tables which were reviewed by the senior member of the investigative team (Tables 1–4 and Supplementary Table S1). Data extracted were the FITT of the ExRx, absolute and relative contraindications to exercise, activities to avoid during exercise, and other special considerations.

**Table 1.** Special Considerations for Pregnant Women Exercising

Professional society	ACOG^3^	ACNM^4^	ACSM^17^	CASEM^22^	Consensus PA Guidelines for Asian Indians^24^	Fitness Australia^23^	FIMS^21^	ODPHP^19^	SASMA^18^	SMA^6^	SOGC/CSEP^7^	PSS^5^	Result
Year	2020	2014	2021	2008	2012	2013	2013	2018	2012	2016	2018	2020	
Know the reasons to stop exercising and consult a health care provider	x	x	x			x	x		x	x	x	x	9
Maintain adequate hydration	x	x	x			x			x	x	x	x	8
Increase caloric intake during prolonged or high-intensity exercise exceeding 45 minutes	x		x			x					x	x	5
Should be under the care of a health care provider	x		x		x	x		x		x			6
Practice controlled breathing and not hold their breath				x	x				x			x	4
Wear supportive and breathable clothing	x		x			x				x		x	5
Some women may not be able to exercise in the third trimester or only at a mild intensity			x		x								2
Women who did not exercise regularly before pregnancy should not start an exercise program until the second trimester				x									1
Contraindications (general)		x				x	x						3
Absolute contraindications			x						x	x	x	x	5
Relative contraindications			x						x	x	x	x	5
Activities to avoid	x	x	x	x	x	x	x	x	x	x	x	x	12
Result	6	4	9	3	4	7	3	2	6	7	6	8	

Fitness Australia specifies that Registered Exercise professionals should know and identify warning signs and instruct their pregnant client(s) to stop exercising rather than pregnant women knowing the warning signs themselves.

An x indicates that the special consideration was listed in the reference.

**Table 2.** Absolute Contraindications to Exercise

Professional society	ACNM^4^	ACSM^17^	Fitness Australia^23^	FIMS^21^	SASMA^18^	SMA^6^	SOGC/CSEP^7^	PSS^5^	Result
Year	2014	2021	2013	2013	2012	2016	2018	2020	
Serious heart disease	x	X		x	x			x	5
Restrictive lung disease	x	X			x			x	4
Hypertension before pregnancy (uncontrolled)	x	x					x	x	4
Preeclampsia	x	x		x	x	x	x	x	7
Premature labor	x	x		x	x	x	x	x	7
Threatened miscarriage	x								1
Incompetent cervix	x	x			x	x	x	x	6
Placenta previa	x	x			x	x	x	x	6
Multiple gestation	x	x			x	x	x	x	6
Unexplained, persistent vaginal bleeding	x	x		x	x		x	x	6
Ruptured membranes	x	x			x	x	x	x	6
Metabolic disorders				x					1
Uncontrolled Type 1 diabetes		x			x		x	x	4
Uncontrolled thyroid disease		x			x		x	x	4
Uncontrolled seizure disorder					x				1
Musculoskeletal condition			x						1
Intrauterine growth restriction		x					x	x	3
Severe anemia		x						x	2
Result	11	14	1	5	11	7	11	14	

Contraindications for ACNM, Fitness Australia, and FIMS were not listed as absolute or relative.

ACOG, CASEM, ODPHP, and Consensus Physical Activity Guidelines for Asian Indians did not provide absolute contraindications to exercise.

An x indicates that the contraindication was listed in the reference.

**Table 3.** Relative Contraindications to Exercise

Professional society	ACSM^17^	SASMA^18^	SMA^6^	SOGC/CSEP^7^	PSS^5^	Result
Year	2021	2012	2016	2018	2020	
Symptomatic anemia	x	x	x	x	x	5
Extreme morbid obesity	x	x	x		x	4
Extreme underweight (BMI <12)	x	x			x	3
Mild/moderate cardiovascular or respiratory disease	x		x	x	x	4
Orthopedic limitations	x	x			x	3
Recurrent pregnancy loss	x			x	x	3
History of spontaneous preterm birth	x		x	x	x	4
Premature labor					x	1
History of premature labor	x					1
History of miscarriage/spontaneous abortion	x		x		x	3
History of fetal growth restriction	x					1
Malnutrition	x		x	x	x	4
Eating disorder	x		x	x	x	4
Twin pregnancy after 28th week			x	x		2
Poorly controlled seizure disorder	x				x	2
History of extremely sedentary lifestyle	x				x	2
Heavy smoker	x				x	2
Poorly controlled Type 1 diabetes mellitus	x		x			2
Poorly controlled hypertension		x	x			2
Poorly controlled hypothyroidism			x			1
Intrauterine growth restriction		x	x			2
Hypertensive disorders of pregnancy/preeclampsia				x		1
Unevaluated maternal cardiac arrhythmia	x	x			x	3
Cervical dilation	x					1
Result	18	7	12	8	15	

ACOG, ACNM, CASEM, Consensus Physical Activity for Asian Indians, FIMS, Fitness Australia, and ODPHP did not provide relative contraindications to exercise.

An x indicates that the contraindication was listed in the reference.

**Table 4.** Activities That Pregnant Women Are Advised to Avoid When Exercising

Professional society	ACOG^3^	ACNM^4^	ACSM^17^	CASEM^22^	Consensus PA Guidelines for Asian Indians^24^	Fitness Australia^23^	FIMS^21^	ODPHP^19^	SASMA^18^	SMA^6^	SOGC/CSEP^7^	PSS^5^	Result
Year	2020	2014	2021	2008	2012	2013	2013	2018	2012	2016	2018	2020	
Contact sports	x	x	x	x		X	x	x	x	x	x	x	11
Supine position after first trimester	x	x	x	x	x	X	x	x		x		x	10
Excessive heat	x	x	x	x		X			x	x	x	x	9
Risk of falling/loss of balance	x	x	x	x			x	x	x	x	x	x	10
Scuba diving	x	x	x				x		x	x	x	x	8
High altitude		x	x	x			x		x	x	x		7
Exercises that cause pain, discomfort, or obstetric symptoms					x	X	x		x				4
Downhill skiing		x	x	x				x	x	x	x		7
Basketball		x	x					x				x	4
Soccer		x	x					x		x		x	5
Ice hockey			x							x	x		3
Nonstationary cycling			x								x	x	3
Abdominal trauma		x		x		X	x	x	x	x			7
Gymnastics		x	x	x					x		x	x	6
Heavy weightlifting/Olympic lifts		x	x			X	x			x	x		6
Horseback riding		x	x	x				x	x	x	x		7
Valsalva maneuver or breath holding			x			x			x		x	x	5
Motionless standing						x			x				2
Excessive abdominal exercises			x	x		x					x	x	5
Stretching beyond comfortable range						x				x			2
Leg exercises that place excessive force on the pubic symphysis										x			1
Vigorous-intensity racquet sports		x							x				2
Free weight training							x						1
Sky diving										x		x	2
Skating									x	x			2
Hang gliding									x				1
Marathon/triathlon							x						1
Exercises that require quick changes in direction			x			x						x	3
Result	6	11	10	11	2	10	10	6	13	13	10	9	

An x indicates that the activity to avoid was listed in the reference.

### Assessment of risk of bias

Two authors (L.R.R. and K.L.) independently assessed report quality using the Appraisal of Guidelines for Research and Evaluation II tool, a validated and widely used instrument in developing and evaluating clinical practice guidelines.^28^ A quality score from 1 (absence of information or the concept is very poorly reported) to 7 (quality of reporting is exceptional and the full criteria and considerations articulated by the tool have been met) was calculated for each of six domains: Scope and Purpose, Stakeholder Involvement, Rigor of Development, Clarity of Presentation, Applicability, and Editorial Independence, and overall assessment scores were tabulated (Table 5). As recommended by the Consortium that developed the AGREE II tool, individual quality scores were used to compare guidelines but a single aggregated quality score was not calculated.^28^ For each domain we grouped the scores using tertile frequency distributions. We categorized “high” quality as a score >85%, “moderate” quality as a score 51%–85%, or “low” quality as a score ≤50% so that guidelines could be compared. When calculating the Applicability domain score for each report, of note, all reports were given a score of 1 (absence of information) for item 20 of the instrument as it was not applicable. For each domain, the mean score, standard deviation (SD), range, and mode were reported.

**Table 5.** AGREE II Risk of Bias Assessment (%)

Professional society	Year	Scope and purpose (1)	Stakeholder involvement (2)	Rigor of development (3)	Clarity of presentation (4)	Applicability (5)	Editorial independence (6)	Overall quality
ACOG^3^	2020	High	Moderate	Moderate	High	High	Low	High
ACNM^4^	2014	Moderate	Low	Low	Moderate	Moderate	Low	Low
ACSM^17^	2021	High	Moderate	Moderate	High	High	Low	High
CASEM^22^	2008	Moderate	Low	Low	Moderate	Low	Low	Moderate
Consensus Physical Activity Guidelines for Asian Indians^24^	2012	High	Moderate	Moderate	Moderate	Low	Low	Moderate
Fitness Australia^23^	2013	High	Moderate	Low	Moderate	Moderate	Low	Moderate
FIMS^21^	2013	High	Low	Low	High	Moderate	Low	Moderate
ODPHP^19^	2018	High	High	High	High	Moderate	Low	Moderate
SASMA^18^	2012	High	Moderate	Low	Moderate	Moderate	Low	Moderate
SMA^6^	2016	High	Moderate	Moderate	High	High	Low	High
SOGC/CSEP^7^	2018	High	High	High	High	High	High	High
PSS^5^	2020	High	Moderate	Moderate	High	Moderate	Low	Moderate
Average ± SD		92.8 ± 6.7	66.7 ± 21.6	59.6 ± 24.0	81.0 ± 12.5	70.1 ± 20.5	34.0 ± 26.8	76.1 ± 14.1

Overall quality is the average of overall quality scores from coders.

ACOG, American College of Obstetrics and Gynecology; ACNM, American College of Nurse Midwives; ACSM, American College of Sports Medicine; CASEM, Canadian Academy of Sports and Exercise Medicine; FIMS, International Federation of Sports Medicine; ODPHP, Office of Disease Prevention and Health Promotion; SASMA, South African Sports Medicine Association; SMA, Sports Medicine Australia; SOGC/CSEP, Society of Obstetricians and Gynecologists in Canada/Canadian Society for Exercise Physiology; PSS, Perinatal Society of Singapore; SD, standard deviation.

### Data synthesis

After summarizing the FITT recommendations, special considerations, contraindications, and activities to avoid during exercise of each qualifying report (L.R.R. and L.D.), the investigative team arrived at a consensus FITT ExRx for women who are pregnant. To produce a consensus FITT ExRx, we made several modifications from the information provided: (1) for intensity, two methods were included in the consensus ExRx: (a) the average recommendation on the Borg 6 (no exertion at all) to 20 (maximal exertion) Rating of Perceived Exertion (RPE) scale^29^- a subjective, self-reported rating of exercise intensity based on the patient's perspective of physical exertion-, and (b) the “talk test,” which is a quick and easy way for women to determine if they are exercising at an intensity that is too high; (2) for time, recommendations for duration per session^4,17,18,21–23^ or accumulated time per week^3,5–7,19,24^ were included, and some reports recommended a number of sets, repetitions, and exercises^6,21,24^ for resistance training; and (3) for type, reports recommended aerobic,^3–7,17–19,21–24^ resistance,^3–7,17–19,21–24^ and flexibility^3–7,17,21–23^ exercise, however, only three recommendations reported neuromotor exercise.^4,5,7^ Characteristics of publications were summarized using SAS 9.4 (SAS Institute, Inc., Cary, NC).

## Results

### Study selection

As shown in Figure 1, the search identified 165 articles, which was reduced to 136 after eliminating duplicates. Seven articles were added from a manual search of the references of included reviews, which totaled 143 articles screened by title. We excluded 80 articles due to the title being about women who were not pregnant, pregnant women with health conditions (*i.e.*, gestational diabetes mellitus, preeclampsia), did not include exercise, or were focused on the outcomes of exercise interventions. The remaining 63 articles were screened by abstract and 23 were excluded because newer recommendations from the same society existed, they focused on exercise outcomes, they were about methodology for developing guidelines, they focused solely on exercising in altitude, or were not guidelines for exercise. The remaining 40 articles were assessed for eligibility by full text, of which 28 were excluded mostly for not being from a professional society as defined above, they did not specify all FITT components, did not have the full text in English, or were reciting the ACOG 2015 guidelines.

### Study characteristics

Of the 12 qualifying articles,^3–7,17–19,21–24^ four professional societies reported recommendations as position statements,^6,18,21,22^ one was a consensus statement,^7^ one was a committee opinion,^3^ two were handouts/packets for clinical utilization,^4,23^ and four were guidelines.^5,17,19,24^ Six reports were published as exercise guidelines during pregnancy,^4,6,17,18,22,23^ four reports were published as physical activity guidelines,^7,19,21,24^ and two reports were published as both exercise and physical activity guidelines during pregnancy.^3,5^ Other information included in the qualifying statements were the benefits of physical activity during pregnancy,^3–7,17–19,21,22,24^ preparticipation health screening,^5,7,17,22,23^ contraindications to exercise,^4–7,17,18,21,23^ activities to avoid,^3–7,17–19,21–24^ and special considerations^3–7,17–19,21–24^ for exercising while pregnant. The methodological approach differed by professional society report with nine societies conducting systematic reviews,^3,5–7,17–19,22,24^ two societies narrating expert opinion and citing quality evidence,^21,23^ and one society not citing methodology or references.^4^ However, only three societies provided a detailed description of the systematic review conducted,^7,19,24^ whereas five societies labeled their report a “review of recent literature” or a similar compatible statement.^3,5,6,18,21^

### Risk of bias

Table 5 describes the methodological quality of included studies. The average overall quality of reports was moderate at 76.1% (SD: 14.1). Overall quality ranged from 50.0% to 100.0% with one report rated low (≤50%), seven reports rated moderate (>50% to ≤85%), and four reports rated high (>85%).

### Synthesis of results

#### Preparticipation health screening

Women were encouraged by the Society of Obstetricians and Gynecologists of Canada/Canadian Society for Exercise Prescription (SOGC/CSEP), Fitness Australia, Canadian Academy of Sport and Exercise Medicine (CASEM), American College of Sports Medicine (ACSM), and Perinatal Society of Singapore (PSS) to complete the Physical Activity Readiness Examination for pregnancy (PARmed-X for pregnancy) with a health care or exercise professional before engaging in an exercise program to identify potential contraindications to exercise.^5,7,17,22,23^ Fitness Australia also identified the Adult Pre-Exercise Screening System as an appropriate option for preparticipation health screening.^23^ Absolute contraindications indicated that exercise would not be appropriate, whereas relative contraindications indicated that exercise should be individualized with guidance from obstetric care providers.^7^ Several professional society recommendations^4–7,17,18,21,23^ listed absolute and relative contraindications to exercise in Tables 2 and 3, respectively, which were somewhat discordant. For example, South African Sports Medicine Association (SASMA) and Sports Medicine Australia (SMA) categorized intrauterine growth restriction as a relative contraindication,^6,18^ whereas ACSM, SOGC/CSEP, and PSS categorized intrauterine growth restriction as an absolute contraindication to exercise.^5,7,17^ Furthermore, the American College of Nurse Midwives (ACNM), Fitness Australia, and International Federation of Sports Medicine (FIMS) did not differentiate between absolute and relative contraindications,^4,21,23^ and ACOG, CASEM, Office of Disease Prevention and Health Promotion's Physical Activity Guidelines of Americans 2nd edition (ODPHP), and Consensus Physical Activity Guidelines for Asian Indians did not specify contraindications to exercise.^3,19,22,24^

#### The FITT of the ExRx

##### Frequency

The most common recommended frequency of exercise was most days of the week. Five reports recommended a minimum of 3 days per week,^3,7,21,22^ four reports recommended daily exercise,^3,6,7,24^ and two reports recommended exercise to be spread throughout the week.^17,19^ Two of the 12 professional societies, SMA and FIMS, specified a frequency of resistance exercise of 2 days per week and 1 to 3 days per week, respectively.^6,21^ SMA recommended a frequency specifically for pelvic floor exercises of 3–4 days per week and ACSM recommended daily pelvic floor exercises.^6,17^

##### Intensity

Moderate-intensity exercise (defined as when a person's heart rate (HR) reaches 40%–59% HR reserve or 64%–76% of their age predicted maximal HR [220-age])^17^ was the intensity recommendation agreed upon by all the professional societies. There was some disagreement on the methods for measuring intensity. ODPHP, CASEM, Consensus Physical Activity Guidelines for Asian Indians, SOGC/CSEP, SMA, PSS, FIMS, ACSM, and ACOG recommended use of the “talk test,”^30^ a subjective measure of exercise intensity during which a person is able to comfortably, with difficulty, or uncomfortably hold a conversation during exercise.^3,5–7,17,19,21,22,24^ SASMA, SMA, ACOG, CASEM, and Fitness Australia recommended that moderate intensity should be determined by an RPE score of 12–14 on the 6 to 20 Borg scale.^3,6,18,22,23^ PSS recommended that moderate intensity should be determined by an RPE score of 13–14 on the 6 to 20 Borg scale.^5^ ODPHP, PSS, and Consensus Physical Activity Guidelines for Asian Indians recommended that moderate intensity should be determined by an RPE score of 5–6 on the 0 to 10 Borg scale.^5,19,24^ PSS, FIMS, ACOG, CASEM, ACNM, SASMA, and SOGC/CSEP suggested intensity should be determined by the age-adjusted maternal HR with an acceptable HR range of 135 to 150 beats per minute for women 20 through 29 years; a HR range of 130 to 145 beats per minute for women 30 through 39 years; and a HR range of 125 to 140 beats per minute for women 40 years and above.^3–5,7,18,21,22^

##### Time

Six professional societies recommended an accumulation of exercise throughout the week^3,5–7,19,24^ and six recommended a duration for each exercise session.^4,17,18,21–23^ Most professional societies agreed that 30 minutes per session was an appropriate duration of exercise. CASEM recommended an upper limit of 40 minutes per session,^22^ SMA and SASMA recommended an upper limit of 60 minutes per session,^6,18^ PSS recommended a lower limit of 20 minutes per session,^5^ and ACSM recommended that any duration of exercise is beneficial.^17^ Fitness Australia suggested to limit exercise session times to prevent overheating and hypoglycemia but did not specify a length of time.^23^ All six professional societies agreed that pregnant women should accumulate 150 minutes per week of exercise,^3,5–7,19,24^ and SMA recommended an upper limit of 300 minutes per week.^6^ Three professional societies indicated a number of sets and repetitions per resistance exercise.^6,21,24^ FIMS provided a more general recommendation of lower weights at higher reps,^21^ whereas SMA and Consensus Physical Activity Guidelines for Asian Indians specified a range of sets and repetitions and/or the number of exercises to perform.^6,24^

##### Type

All 12 professional societies agreed that aerobic exercise was the primary form of exercise, but also recommended resistance exercise for pregnant women. All professional societies, except for ODPHP, described in further detail what kind of aerobic exercise was safe and effective for pregnant women.^3–7,17,18,21–24^ These aerobic activities included both weight and nonweight-bearing exercises, such as walking, jogging, running, swimming, hiking, stationary cycling, cross-country skiing, dancing, rowing, water aerobics/aquafit, and low-impact aerobics. ACNM, ACOG, Consensus Physical Activity Guidelines for Asian Indians, FIMS, SASMA, SMA, and PSS described in further detail what kind of resistance exercise was safe and effective for pregnant women.^3–6,18,21,24^ These resistance activities included both weight and nonweight-bearing exercises using light free weights, machines, resistance and elastic bands, and body weight targeting all major muscle groups. SOCG/CSEP, ACNM, and PSS recommended neuromotor exercises, such as yoga and Pilates, but only the PSS specified that these activities should be modified.^4,5,7^ ACSM, ACNM, SOGC/CSEP, FIMS, ACOG, SMA, PSS, CASEM, and Fitness Australia recommended flexibility exercises mostly for purposes of warm up and cool down surrounding exercise sessions.^3–7,17,21–23^

#### Special considerations

The special considerations are displayed in Table 1 and activities that pregnant women should avoid in Table 4. The SOGC/CSEP and SASMA did not list lying on back after first trimester as a special consideration, but advised women who experience light-headedness, nausea, or feel unwell when exercising while lying flat on their back to modify their exercise position and avoid the supine position.^7,18^ SOGC/CSEP rated this recommendation as weak due to very low-quality evidence gathered on this topic during their systematic review of the literature,^7^ and SASMA similarly stated that this recommendation is controversial due to conflicting existing evidence.^18^ The Consensus Physical Activity Guidelines for Asian Indians stands alone in not listing physical contact sports as an activity to avoid.^24^

There is more consistency observed between the professional societies among the activities to avoid than with the special considerations. CASEM stands alone in suggesting that women who did not previously engage in exercise before their pregnancy should not start an exercise program until the second trimester.^22^ In contrast, the other 11 professional societies stated that beginning an exercise routine at any time during pregnancy can be safe and effective.

## Discussion

### Main findings

**FIG. 2.**
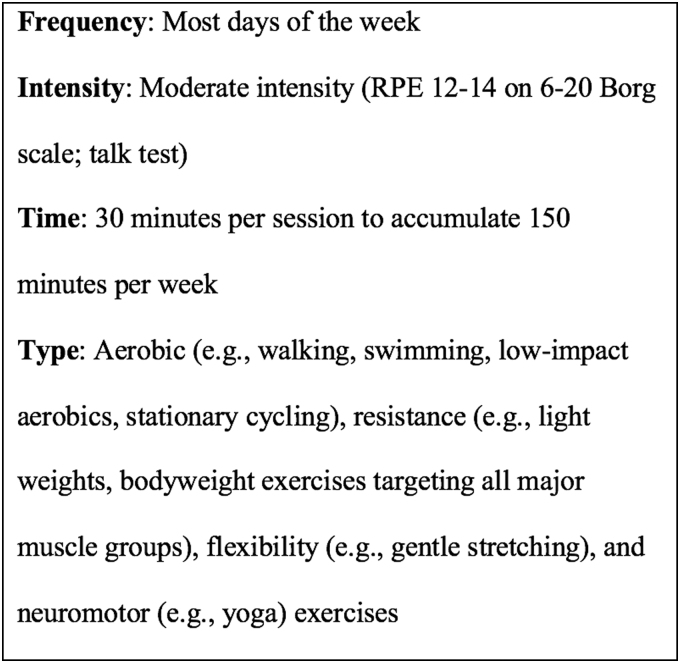
Consensus exercise prescription for healthy women who are pregnant.

Because professional society guidelines varied, we conducted a systematic review to assemble a consensus FITT ExRx for healthy pregnant women without exercise contraindications so that prenatal care providers can better advise their patients and encourage safe exercise during pregnancy for its numerous health benefits for both mother and baby. Due to our comprehensive search of the literature adhering to PRISMA standards, we have provided prenatal care providers with more informed guidance when recommending exercise to their patients. Across professional society guidelines, the consensus FITT ExRx for women who are pregnant without exercise contraindications is summarized in Figure 2 and is as follows: frequency of most days of the week, moderate-intensity (RPE of 12–14 on the 6 to 20 Borg scale and/or *via* the talk test), 30-minute exercise sessions to accumulate 150 minutes of exercise per week. Exercise type should include aerobic (*e.g.*, walking, swimming, low-impact aerobics, stationary cycling), resistance (*e.g.*, light weights, bodyweight exercises targeting all major muscle groups), flexibility (*e.g.*, gentle stretching), and neuromotor (*e.g.*, yoga). Women who are pregnant, especially those who were previously physically inactive, should progress slowly and gradually increase the frequency and time components of their exercise plan.

This systematic review included an evaluation of the quality of the existing professional society recommendations for exercise during pregnancy using the AGREE II tool.^28^ All 12 professional society guidelines described their scope and purpose well (average quality rating 93%) and presented information clearly (average quality rating 81%). However, the scores for the remaining domains indicated areas of improvement for future versions of professional society guidelines as follows: stakeholder involvement (average quality rating 67%) reflecting patient views and preferences; rigor of development (average quality rating 60%) describing the criteria used for selecting evidence and methods for formulating recommendations, utilizing external review before publication, and providing procedures for updating guidelines; applicability (average quality rating 70%) reflecting potential resource implications of applying the recommendations, and facilitators and barriers to their application; and editorial independence (average quality rating 34%) showing the transparency of editorial independence and conflicts of interest. It is important to note that one of the 12 professional society recommendations, SOGC/CSEP, used the AGREE II tool as a guide for the development of their guidelines.^7^

Furthermore, we tabulated the common contraindications and special considerations. Across the 12 professional society guidelines, the most common absolute contraindications were preeclampsia (58%), premature labor (58%), ruptured membranes (50%), placenta previa (50%), incompetent cervix (50%), unexplained, persistent vaginal bleeding (50%), and multiple gestation (50%); whereas the most common relative contraindications were symptomatic anemia (42%), extreme morbid obesity (33%), mild-to-moderate cardiovascular or respiratory disease (33%), history of spontaneous preterm birth (33%), malnutrition (33%), and diagnosis of an eating disorder (33%). Common physical activities to avoid included physical contact sports (92%), supine position after the first trimester (83%), activities with a danger of falling or loss of balance (83%), excessive heat (75%), and scuba diving (66%). Finally, the most common special considerations were the importance of knowing the reasons to stop exercising and consult a health care provider (75%), maintaining adequate hydration (66%), and continuing prenatal care under a health care provider who can monitor the progress of the pregnancy (50%).

Our findings have additional important implications for clinical practice. Currently, many prenatal care providers do not routinely provide exercise advice to their patients.^9,31^ When providers do offer exercise advice, pregnant women often perceive provider knowledge as limited due to a lack of explicit recommendations.^32^ This lack of confidence by both prenatal providers and their patients regarding being physically active during pregnancy is partially due to conflicting advice from health care providers and/or the variability in the professional recommendations themselves.^33,34^ The consensus FITT ExRx consolidated from our review can better inform clinicians and lessen their concerns about advising their patients to exercise safely during pregnancy and provide their patients without exercise contraindications the confidence to exercise during pregnancy for its many health benefits for both the mother and baby.

### Comparison with existing literature

To our knowledge, this is the first systematic review to examine physical activity recommendations for healthy pregnant women framed by the FITT principle of ExRx utilizing the AGREE II tool for assessment of guideline quality.^28^ Previous systematic reviews that evaluated exercise recommendations for healthy pregnant women included clinical trials,^35,36^ and some have solely evaluated clinical practice guidelines to produce a consensus recommendation for physical activity during pregnancy.^37–41^ Interestingly, the recommendations that the systematic reviews of clinical trials arrived at are relatively consistent with the consensus FITT ExRx we compiled. For example, a review in 2016 concluded that previously sedentary pregnant women should exercise 3 to 4 times per week at an appropriate HR zone or RPE, and/or using the talk test, starting at 15 minutes and progressing to 30-minute sessions, incorporating aerobic exercises that move large muscle groups such as walking, swimming, and cycling.^35^ Similarly, another recent systematic review of randomized controlled trials (*K* = 19) examined the effects of physical activity on maternal and fetal outcomes and concluded that women who are pregnant should exercise 3 to 4 times per week at an intensity equivalent to a HR at 60–80% of aerobic capacity or 12–14 on the Borg RPE scale and/or using the talk test, starting at 15 minutes then progressing to 30 minutes per session, incorporating aerobic and resistance training of large muscle groups.^36^

Consensus statements and systematic reviews summarizing physical activity recommendations during pregnancy all agree an international, updated consensus regarding appropriate exercise during pregnancy would benefit pregnant women by promoting a healthy lifestyle while ensuring safety during exercise.^37^ However, it is important to note that these peer-reviewed articles were often narrative versus systematic reviews, included clinical populations excluded from our systematic review, and/or included guidelines not framed by the FITT principle of ExRx. As such, one consensus statement published by the International Olympic Committee provided recommendations for recreational and elite athletes during pregnancy with a focus on contraindications and special considerations but did not provide recommendations for the duration, time, or type of exercise other than general aerobic and resistance training.^38^ Artral summarized and expanded on the rationale supporting the 2015 ACOG Consensus Statement and provided additional recommendations for pregnant women with obesity or at risk for gestational diabetes mellitus, preeclampsia, and fetal macrosomia, clinical populations not included in our systematic review.^39^ Of note, this review referenced three professional society recommendations included in our review, ODPHP, ACOG published in 2015, and ACSM, and our review expands on these findings.^39^

Evenson et al. conducted a similar review of professional society, peer-reviewed guidelines published between 1990 and 2012.^40^ This systematic review of guidelines was similar to ours in arriving at the conclusion of performing moderate-intensity exercise during pregnancy.^40^ However, Evenson et al. tabulated the number of guidelines that reported a frequency, time, and type of exercise appropriate for pregnant women but did not provide the specifics of these components of the ExRx.^40^ Our review includes updated versions of the guidelines critiqued by Evenson et al. and provides a consensus FITT ExRx for women who are pregnant. Furthermore, the scope of the review by Evenson et al. (2013) was limited to PubMed, whereas our review searched four other databases in addition to PubMed to include: 11 guidelines in Evenson et al. and 12 in our review.^40^

In 2019, Evenson et al. published another report summarizing physical activity recommendations during pregnancy from three national guidelines, the ACOG published in 2015, ODPHP, and SOGC/CSEP, and one international guideline, the International Olympic Committee.^41^ This review provided a consensus ExRx that is consistent with the consensus FITT ExRx we have compiled; at least 150 minutes spread throughout the week or at least 20 to 30 minutes per day of moderate-intensity physical activity using both aerobic and muscle conditioning activities.^41^ Our review expands on these findings to include all four components of the FITT principle and critiques a greater number of professional society guidelines to produce a consensus ExRx that is more representative of current recommendations.

Tsakiridis et al. published a comparative review of three exercise guidelines during pregnancy: ACOG published in 2015, SOGC published in 2018, and the Royal Australian and New Zealand College of Obstetricians and Gynecologists.^37^ This review summarized each professional society's recommendations but did not provide a consensus ExRx.^37^ Our review, in contrast, utilized updated guidelines from ACOG and SOGC along with several other guidelines to produce a consensus ExRx while considering the quality of each set of recommendations. The consensus FITT ExRx we consolidated now provides women who are pregnant with more exercise options and variety which may translate to more women choosing to stay physically active during pregnancy.

### Strengths and limitations

This systemic review has limitations. It is important to note that we did not search gray literature and limited our database search to PubMed, Scopus, SPORTDiscus, CINAHL, and the Cochrane library raising the possibility that professional guidelines could have been missed. Also, only two qualifying reports included recommendations for resistance training frequency, sets, and repetitions, which did not allow for a consensus to be reached regarding this exercise modality.

A strength of this review is we evaluated the most current professional society recommendations available with qualifying reports ranging in publication date from 2008 to 2021. Another strength is we tallied the most common FITT ExRx recommendations, absolute and relative contraindications, and special considerations for clinicians to consider advising their patients to exercise. The use of the AGREE II tool to evaluate the professional society exercise recommendations was beneficial as this tool is often cited for development of clinical practice guidelines. Current research and guidelines do not indicate an upper limit of exercise while pregnant, which may result in pregnant women refraining from exercising at exercise intensities that could be interpreted as vigorous. Further research in this field should provide better informed guidance relating to safety concerns for women who are pregnant, particularly regarding upper limits of exercise intensity. A recent guideline published by the European Society of Cardiology stated pregnant athletes may continue to train intensively during pregnancy but not exceed a HR of >90% of age-predicted maximum,^42^ but more research is needed to confirm this recommendation.

## Conclusion

This systematic review of exercise recommendations from 12 professional societies produced a consensus FITT ExRx for women who are pregnant without exercise contraindications to help prenatal care providers and their pregnant patients feel comfortable with women undertaking an exercise program during pregnancy. Furthermore, our findings from the AGREE II tool provide professional societies with methodological guidance for updates of these guidelines. More research is needed to determine the upper limits of exercise for women who are pregnant who wish to continue exercising vigorously during pregnancy and to determine an appropriate ExRx for resistance training during pregnancy.

## Supplementary Material

Supplemental data

Supplemental data

## Abbreviations Used

ACNM: American College of Nurse Midwives
ACOG: American College of Obstetrics and Gynecology
ACSM: American College of Sports Medicine
CASEM: Canadian Academy of Sport and Exercise Medicine
CINAHL: Cumulative Index of Nursing and Allied Health Literature
ExRx: exercise prescription
FIMS: International Federation of Sports Medicine
FITT: Frequency, Intensity, Time, and Type
HR: heart rate
ODPHP: Office of Disease Prevention and Health Promotion
PARmed-X for pregnancy: Physical Activity Readiness Examination for pregnancy
PRISMA: Preferred Reporting Items for Systematic Reviews and Meta-Analyses
PSS: Perinatal Society of Singapore
RPE: Rating of Perceived Exertion
SASMA: South African Sports Medicine Association
SD: standard deviation
SMA: Sports Medicine Australia
SOGC/CSEP: Society of Obstetricians and Gynecologists of Canada/Canadian Society for Exercise Prescription
