# Supplementary material for: Professional Exercise Recommendations for Healthy Women Who Are Pregnant: A Systematic Review
Source: Womens Health Rep (New Rochelle). 2021 Sep 20;2(1):400–12. doi: 10.1089/whr.2021.0077 (PMC8524738; doi:10.1089/whr.2021.0077)
Supplement: Supplemental data [file Supp_Data.docx]

PubMed Search

("exercise"[majr] OR “exercise”[ti] OR exercises[ti] OR exercising[ti] OR postexercise[ti] OR running[mesh] OR running[ti] OR aerobics OR bicycling[mesh] OR bicycling OR bicycle* OR cycling[ti] OR treadmill* OR ergometer* OR "weight lifting" OR "weight training" OR "resistance training" OR "strength training" OR "endurance training" OR "speed training" OR "circuit training" OR “physical activity” OR “yoga” OR “pilates” OR “tai chi” OR Taichi[tiab] OR "T'ai Chi" OR "Tai Ji" OR "Tai-Ji" OR “Taijiquan” OR "training duration" OR "training frequency" OR "training intensity" OR "aerobic endurance" OR "aerobic training" OR "interval training" OR "combination training" OR "combined training" OR plyometric* OR "HIIT" OR walking[mesh] OR walking[ti] OR swimming OR “Exercise Movement Techniques"[Mesh]) AND (pregnanc* OR pregnant OR gestation* OR "Pregnancy"[Mesh] OR primipara* OR multipara*) AND (guideline*[ti] OR statement*[ti] OR “position stand”[ti] OR recommendations[ti]) AND English[la] NOT ((animals[mesh] NOT humans[mesh]) OR Clinical Trial[ptyp] OR Comparative Study[ptyp] OR Controlled Clinical Trial[ptyp] OR Clinical Study[ptyp] OR "Diet Therapy"[Mesh] OR "diet therapy" [Subheading] OR diet[ti] OR nutrition[ti] OR dietary[ti] OR "Diet"[Mesh] OR "Nutritional Status"[Mesh])

Other Database Searches

Three searches were run independent of one another. They were combined using the search history:

(S1 AND S2) NOT S3

**Search 1:**

Line 1 (in title): exercise OR exercises OR exercising OR postexercise OR running OR cycling OR walking

OR

Line 2: aerobics OR bicycling OR bicycling OR bicycle* OR treadmill* OR ergometer* OR "weight lifting" OR "weight training" OR "resistance training" OR "strength training" OR "endurance training" OR "speed training" OR "circuit training" OR “physical activity” OR “yoga” OR “pilates” OR “tai chi” OR Taichi OR "T'ai Chi" OR "Tai Ji" OR "Tai-Ji" OR “Taijiquan” OR "training duration" OR "training frequency" OR "training intensity" OR "aerobic endurance" OR "aerobic training" OR "interval training" OR "combination training" OR "combined training" OR plyometric* OR "HIIT" OR swimming

**Search 2:**

Line 1: pregnanc* OR pregnant OR gestation* OR primipara* OR multipara*

AND

Line 2 (in title): guideline* OR statement* OR “position stand” OR recommendations

**Search 3:**

Line 1 (in title): “Clinical Trial” OR “Comparative Study” OR “Clinical Study” OR diet OR nutrition* OR dietar*
