# Supplementary material for: Professional Exercise Recommendations for Healthy Women Who Are Pregnant: A Systematic Review
Source: Womens Health Rep (New Rochelle). 2021 Sep 20;2(1):400–12. doi: 10.1089/whr.2021.0077 (PMC8524738; doi:10.1089/whr.2021.0077)
Supplement: Supplemental data [file Supp_TableS1.docx]

Exercise Prescription Guidelines, Scientific Statements, and Recommendations for Healthy Pregnant Women by Various Professional Societies

| Professional Society | Year | Frequency | Intensity | Time | Type |
| --- | --- | --- | --- | --- | --- |
| American College of Sports Mediicne^17^ | 2021 | Throughout the week  Pelvic floor muscle training: daily | Moderate defined as a woman being able to maintain a conversation during exercise and measured via the talk test | 20-30min/session | Aerobic: walking, swimming, stationary cycling, low-impact aerobics, running  Resistance  Pelvic floor muscle training: Kegel exercises  Flexibility: stretching, warm up/cool down |
| American College of Nurse Midwives^4^ | 2014 | Most days of the week | Moderate defined as start to sweat and increased HR, but still able to talk | At least 30 min/session | Aerobic: walking, swimming, biking, low impact aerobics, hiking, dancing, rowing  Resistance: light weight training  Neuromotor: yoga  Flexibility: stretching, warm up/cool down |
| American College of Obstetricians and Gynecologists^3^ | 2020 | Most or all days of the week; at least 3-4 d/wk up to daily | Moderate, RPE 12-14 (Borg 6-20 scale); talk test; less than 60-80% predicted maximum maternal HR | 150 min/wk  At least 20-30min/session | Aerobic: walking, stationary cycling, low-impact aerobics, dancing, hydrotherapy/water aerobics  Resistance: strength training using weights and elastic bands  Flexibility: stretching |
| Canadian Academy of Sports and Exercise Medicine^22^ | 2008 | 3d/wk  Maximum 4-5d/wk | Moderate, RPE 12-14 (Borg 6-20 scale); talk test | Maximum 30-40min/session | Aerobic: less strenuous but continuous (brisk walking, stationary cycling, cross-country skiing, swimming)  Resistance: strength conditioning  Flexibility: stretching, warm up/cool down |
| Consensus Physical Activity Guidelines for Asian Indians^24^ | 2012 | Most, if not all, days of the week | Moderate, RPE 5-6 (Borg 0-10 scale); talk test | Aerobic: at least 30 min/session to accumulate 150min/wk  Resistance: moderate-to-high reps (1-2 sets of 12-15 reps) | Aerobic: walking, cycling, swimming  Resistance: light free weights, machines, elastic bands, calisthenics  Pelvic floor strengthening |
| International Federation of Sports Medicine^21^ | 2013 | 3-4 d/wk for non-physically active  Resistance: 1-3 d/wk | Endurance training monitored by individually adjusted HR by age (20-29yrs 135-150 HR; 30-39yrs 130-145 HR; >40yrs 125-140 HR); talk test | Aerobic: 30 min/d for non-physically active  Resistance: lower weights at higher reps of 8-10 exercises | Aerobic: cycling, swimming, hiking, walking, jogging, Nordic walking, cross-country skiing  Resistance: light weights (no free weights)  Flexibility: stretching |
| Fitness Australia^23^ | 2013 | Most days of the week | Moderate, RPE 12-14 (Borg 6-20 scale) | Limit session times to avoid hypoglycemia and overheating;  longer bouts of low intensity | Aerobic: Low impact aerobics, water aerobics  Resistance: general and pelvic floor, core, and postural strengthening  Flexibility: stretching within comfortable range, warm up/cool down |
| Office of Disease Prevention and Health Promotion^19^ | 2018 | Spread throughout the week | Light to moderate, RPE 5-6 (Borg 0-10 scale); talk test | At least 150 min/wk | Aerobic  Resistance: muscle strengthening |
| Society of Obstetricians and Gynecologists in Canada/Canadian Society for Exercise Physiology ^7^ | 2018 | At least 3d/wk but daily is encouraged | Moderate, measured by maternal HR and/or talk test | At least 150 min/wk | Aerobic: brisk walking, stationary cycling, swimming, aquafit  Resistance  Pelvic floor strengthening: Kegel exercises  Flexibility: gentle stretching, warm up/cool down  Neuromotor: yoga |
| South African Sports Medicine Association^18^ | 2012 | Most or all days of the week | Moderate, RPE 12-14 (Borg 6-20 scale); maintain HR between 55-70% of predicated maximum | 30-60 min/d | Aerobic: walking, jogging, running, hiking, low-impact aerobics, swimming, stationary cycling, rowing, cross country skiing, dancing  Resistance: low weight and dynamic range of motions, conditioning exercises |
| Sports Medicine Australia^6^ | 2016 | Most if not all days of the week  Aerobic: daily  Resistance: 2 d/wk  Pelvic floor exercises: 3-4d/wk | Aerobic: Moderate, RPE 12-14 (Borg 6-20 scale); talk test  Muscle strengthening: submaximal intensity  Pelvic floor exercises: contract ‘maximally’ | Aerobic: 150-300 min/wk moderate or 75 min/wk vigorous (30-60 min/d)  Resistance: 1 set of 12-15 reps of up to 8-10 exercises  Pelvic floor exercises: squeeze for 4 to 30 second holds; mixture of slow and controlled and fast and controlled | Aerobic: brisk walking, running, jogging, stationary cycling, swimming, aerobics  Resistance: body weight, light weights, and/or resistance bands; work all large muscle groups  Pelvic floor exercises: sit with weight forward (hands on knees) and sitting upright; sitting, kneeling, standing, lying down, or standing with legs astride position  Flexibility: stretching, warm up/cool down |
| Perinatal Society of Singapore^5^ | 2020 | At least 3 d/wk, ideally most days of the week | Moderate, RPE 13-14 (Borg 6-20 scale); RPE 5-6 (Borg 0-10 scale); talk test; target maternal HR | At least 150 min/wk  At least 20-30min/d  15 min/d for highly inactive women then progress | Aerobic: brisk walking, swimming, stationary cycling, low-impact aerobics, jogging  Resistance: body weight exercises such as squats, lunges, pushups; light dumbbells/weights, resistance band exercises  Neuromotor: modified yoga, modified Pilates  Flexibility: stretching, warm up/cool down |
| Consensus |  | Most days of the week | Moderate, RPE 12-14 (Borg 6-20 scale); talk test | 150min/wk  30min/d | Aerobic: walking, swimming, low-impact aerobics, stationary cycling  Resistance: light weights, bodyweight exercises; target all major muscle groups  Flexibility: gentle stretching, warm up/cool down  Neuromotor: yoga |

RPE= Rating of Perceived Exertion

d=day(s); wk=week; min=minutes; / =per

reps=repetitions
